# Using gaze patterns to predict task intent in collaboration

**DOI:** 10.3389/fpsyg.2015.01049

**Published:** 2015-07-24

**Authors:** Chien-Ming Huang, Sean Andrist, Allison Sauppé, Bilge Mutlu

**Affiliations:** Department of Computer Sciences, University of Wisconsin–MadisonMadison, WI, USA

**Keywords:** intention, eye gaze, support vector machine, gaze patterns, intention prediction

## Abstract

In everyday interactions, humans naturally exhibit behavioral cues, such as gaze and head movements, that signal their intentions while interpreting the behavioral cues of others to predict their intentions. Such intention prediction enables each partner to adapt their behaviors to the intent of others, serving a critical role in joint action where parties work together to achieve a common goal. Among behavioral cues, eye gaze is particularly important in understanding a person's attention and intention. In this work, we seek to quantify how gaze patterns may indicate a person's intention. Our investigation was contextualized in a dyadic sandwich-making scenario in which a “worker” prepared a sandwich by adding ingredients requested by a “customer.” In this context, we investigated the extent to which the customers' gaze cues serve as predictors of which ingredients they intend to request. Predictive features were derived to represent characteristics of the customers' gaze patterns. We developed a support vector machine-based (SVM-based) model that achieved 76% accuracy in predicting the customers' intended requests based solely on gaze features. Moreover, the predictor made correct predictions approximately 1.8 s before the spoken request from the customer. We further analyzed several episodes of interactions from our data to develop a deeper understanding of the scenarios where our predictor succeeded and failed in making correct predictions. These analyses revealed additional gaze patterns that may be leveraged to improve intention prediction. This work highlights gaze cues as a significant resource for understanding human intentions and informs the design of real-time recognizers of user intention for intelligent systems, such as assistive robots and ubiquitous devices, that may enable more complex capabilities and improved user experience.

## 1. Introduction

In daily interactions, humans frequently engage in *joint action*—a collaborative process that involves parties working together to coordinate attention, communication, and actions to achieve a common goal (Clark, 1996; Sebanz et al., 2006). For example, movers carrying a large piece of furniture, an instructor training students in a chemistry lab, or a server taking customer orders at a deli counter must coordinate their behaviors with one another. To achieve successful joint action, people monitor each others' actions and task progress, predict each others' intentions, and adjust their own actions accordingly (Sebanz and Knoblich, 2009). Such action monitoring and intention prediction are integral to the establishment of common ground between parties engaged in joint action. As a result, parties consciously and subconsciously exhibit behavioral cues, such as eye gaze and gestures, to manifest intentions for others to read while interpreting others' behavioral cues to understand their intention, thereby facilitating joint action. These behavioral cues are a gateway to understanding a person's mental states, including attention, intentions, and goals. Moreover, increasing evidence from neuroscience and developmental psychology has shown that action monitoring allows people to use their behavior repertoire and motor system to predict and understand others' actions and intentions (Blakemore and Decety, 2001; Buccino et al., 2001; Rizzolatti and Craighero, 2004).

Among other behaviors, gaze cues are particularly informative in the manifestation of mental states. Deictic gaze toward an object, for instance, may signal the person's interest in the object and has been found to be temporally coupled with the corresponding speech reference to the object (Meyer et al., 1998; Griffin, 2001). Moreover, people use gaze cues to draw others' attention toward an intended object in the environment in order to establish perceptual common ground (Sebanz et al., 2006). The ability to understand and follow such cues is critical for sharing mental states in an interaction (Butterworth, 1991). Gaze cues may also signal planned actions; empirical evidence has shown that gaze cues indicate action intent and lead motor actions that follow (Land et al., 1999; Johansson et al., 2001).

While prior research has highlighted the link between gaze cues and intention, the current work aims to develop a model quantifying how patterns of gaze cues may characterize and even predict intentions. To this end, we collected data of dyadic interactions in which a “customer” and a “worker” engaged in a sandwich-making task and analyzed how the customers' gaze patterns indicated their intentions, which we characterized as the ingredients they chose. Conceptually, this interaction can be characterized as involving three processes: (1) the customer looks at possible ingredients to make a decision about which ingredient to request (Hayhoe and Ballard, 2014); (2) the customer signals their decision via behavioral cues (Pezzulo et al., 2013); and (3) the worker observes the customer's gaze behaviors to predict their intentions (Doshi and Trivedi, 2009; Ognibene and Demiris, 2013; Ognibene et al., 2013). Our goal is to quantify how much information the customer's gaze provides about their intentions in the first two processes. We built and tested a machine learning model that predicted customer intentions from tracked eye gaze data. Specifically, we developed a support vector machine-based approach that predicted the customers' intention—choice of ingredients—based on their exhibited gaze patterns. The effectiveness of the predictor was evaluated using the collected gaze data. Our model and findings contribute to our understanding of the relationship between gaze cues and intent and to design guidelines for emerging technologies, such as assistive robots and ubiquitous devices, that utilize real-time intention prediction to provide their users with effective and anticipatory assistance.

This paper is organized as follows. Section 2 reviews behavioral signals of human intentions and action monitoring for intention understanding. We present a computational model that quantifies the relationship between gaze cues and human intentions and an evaluation of the effectiveness of the model in Section 3. We discuss our results, potential applications, and limitations of this work in Section 4.

## 2. Background

In everyday interactions, from carrying furniture to successfully navigating in a crowded space, people engage in an implicit form of coordination (Sebanz et al., 2006). This coordination relies on the successful communication and recognition of intent by the parties involved in the interaction and enables each person to adapt their behavior to accommodate their partner's intentions. While communicating intent can be achieved through a number of behavioral channels (Morris and Desebrock, 1977; White, 1989; Clark and Brennan, 1991; Shibata et al., 1995; Bangerter, 2004), gaze has been identified as crucial in understanding the intentions of others, as the direction of gaze indicates where a person is directing their attention and the actions that they may subsequently perform (Baron-Cohen et al., 2001; Meltzoff and Brooks, 2001). Below, we review research into how humans develop an understanding of intent in themselves and others and utilize gaze cues to communicate intent.

### 2.1. Human intent

The concept of intentionality is defined as the commitment of a person to executing a particular action (Malle and Knobe, 1997). The formulation of an intent is often driven by the individual's desire to achieve a particular goal (Astington, 1993). This formulation requires a variety of other skills, including forethought and planning, to appropriately fulfill an intention (Bratman, 1987). What differentiates an intent from a desire is this level of planning in preparation to turn the intention into an achievable reality (d'Andrade, 1987).

From an early age, children begin to attribute intent to the actions of others. For example, children at 15 months of age are capable of understanding the intentions of others in physical tasks, even when the goal is not achieved (Meltzoff, 1995). Later, children learn how behaviors are driven by intent (Feinfield et al., 1999), contributing to the development of an ethical system where intentionality is used as a factor to establish the culpability of an individual.

Prior work suggests that, after developing a capacity for understanding intent, humans also develop *Theory of Mind* (ToM)—the ability to attribute mental states to others (Leslie, 1987). The development of ToM enables people to understand that other humans they interact with may have intents that can differ from their own (Leslie, 1987; Blakemore and Decety, 2001). ToM then shapes the way people interact with one another in a way that is most easily observable in physical tasks, such as moving a table together or navigating through a crowd. In these scenarios, humans rely on ToM abilities to attribute intent to other participants and to adapt their own behaviors to accommodate the intent of others, resulting in seamless interactions.

### 2.2. Communicating intent via gaze

While the ability to attribute intent to others is important in joint action, discerning what the intentions of other participants are with a high degree of reliability can be difficult without some amount of evidence. One approach people subconsciously use to infer the intent of others is by observing their behavioral cues (Blakemore and Decety, 2001). Humans employ a number of behavioral cues, such as gaze and gestures, when working with others on a task (Morris and Desebrock, 1977; White, 1989; Clark and Brennan, 1991; Shibata et al., 1995; Baron-Cohen et al., 2001; Meltzoff and Brooks, 2001; Bangerter, 2004). These cues aid in their partner's understanding of and fluency in the task, enabling their partner to adjust their behavior accordingly to accommodate intended actions (Blakemore and Decety, 2001). While a number of behavioral channels can be used to understand intent, gaze is considered preeminent among them due to the clarity with which it can indicate attention; for instance, partners would assume that an area being gazed toward will be the next space to be acted upon (Baron-Cohen et al., 2001; Meltzoff and Brooks, 2001).

Gaze behavior is crucial to human communication of intent throughout the development of social behavior. During infancy, children can follow the gaze cues of adults, which serve as the basis of joint attention (Butler et al., 2000), and use their own gaze to communicate an object of interest (Morales et al., 1998). Older preverbal children can employ gaze in conjunction with gestures to communicate more concretely (Masur, 1983). The use and understanding of gaze becomes more complex and nuanced with age, allowing humans to better identify targets of joint attention (Heal, 2005). This development of gaze understanding mirrors the development of understanding of intent and ToM discussed above, allowing humans to gradually develop a more complex intuition of others and their intentions.

During an interaction, gaze behavior can indicate one's intent in a variety of ways, such as communicating a future action or an emotional state. During a joint task, awareness of a partner's gaze behavior helps enable effective task coordination between participants (Tomasello, 1995). Prior work by Brennan et al. (2008) used head-mounted eye trackers to examine gaze patterns during a joint search task. Awareness of a partner's gaze behavior was not only sufficient for completing the task, but it also resulted in significantly faster search times than verbal coordination did. Additionally, participants who were aware of their partner's gaze behavior offered more precise help during the task when it was necessary. Adams and Kleck (2005) conducted a controlled laboratory study where participants were presented with photographs of people who were either gazing toward or away from the participant. Results showed that participants' perceptions of the photographed person's emotional state were affected by the person's gaze direction.

Gaze behavior can be used in conjunction with other attributes or behavioral cues to more accurately predict intent. Ordering of gaze fixations has been used to infer the type of visual task a person is performing, such as memorizing a picture vs. counting the number of people photographed in a picture (Haji-Abolhassani and Clark, 2014). Prior work used eye gaze and its associated head movements as input for a sparse Bayesian learning model (McCall et al., 2007) to predict a driver's future actions when operating a motor vehicle (Doshi and Trivedi, 2009). Additionally, work by Yi and Ballard (2009) built a dynamic Bayesian network from a user's gaze and hand movements to predict their task state in real time during a sandwich-building task.

While prior work has examined the connection between gaze and intent in a variety of situations, the current work aims to provide an empirical approach to modeling gaze behavior to predict task intent during collaboration. Specifically, it extends prior work in two ways. First, the current work investigates the relationship between gaze cues and task intent in a collaborative context, whereas prior work employed tasks that involved only one person completing them, e.g., making a sandwich (Yi and Ballard, 2009) or driving a car (Doshi and Trivedi, 2009). Second, the prior predictive models utilized multiple sources of information, while this present work focuses on using gaze cues only. A related problem to the focus of the present work is how to use the predicted intention of others to direct one's own focus (e.g., gaze fixation). For example, Ognibene and Demiris (2013) and Ognibene et al. (2013) utilized people's motions to predict their intentions and used these predictions to control the attention of a robotic observer.

## 3. Prediction of human intentions

In this section, we describe our process for understanding and quantifying the relationship between gaze cues and human intentions. This process includes collecting human interaction data, modeling the characteristics of gaze patterns from our data, and evaluating the effectiveness of the computational model. In addition to the quantitative evaluation, we provide qualitative analyses of the circumstances under which our model succeeds and fails in predicting user intentions.

### 3.1. Data collection and annotation

**Figure 1:**
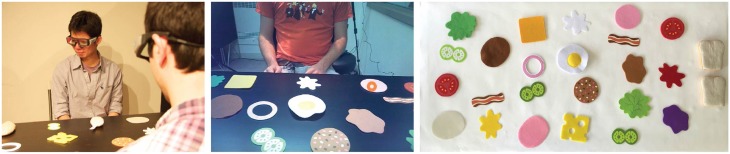
**Data collection of dyadic interactions in a sandwich-making task. Left:** Two participants, wearing gaze trackers, working together to make a sandwich. **Middle**: The participant's view of the task space from the gaze tracker. The orange circle indicates their current gaze target. **Right**: The layout of ingredients on the table. The ingredients, from top to bottom, left to right, are *lettuce1, pickle1, tomato2, turkey, roast beef, bacon2, mustard, cheddar cheese, onions, pickle2, ham, mayo, egg, salami, swiss cheese, bologna, bacon1, peanut butter, lettuce2, pickle3, tomato1, ketchup, jelly*.

Our data collection involved pairs of human participants engaged in a collaborative task. We used this study both to collect data for our model as well as to build an intuition as to how joint attention is coordinated through both verbal and non-verbal cues in day-to-day human interactions. During the data collection study, participants performed a sandwich-making task in which they sat across from each other at a table that contained 23 possible sandwich ingredients and two slices of bread. The initial layout of the ingredients was the same for each pair of participants (Figure 1). One participant was assigned the role of “customer,” and the other was assigned the role of “worker.” The customer used verbal instructions to communicate to the worker what ingredients he/she wanted on the sandwich. Upon hearing the request from the customer, the worker immediately picked up that ingredient and placed it on top of the bread.

We recruited 13 dyads of participants for the data collection study. All dyads were recruited from the University of Wisconsin–Madison campus and were previously unacquainted. The protocol for the data collection study was reviewed and approved by the University of Wisconsin–Madison's Education and Social/Behavioral Science Institutional Review Board (IRB). Prior to the experiment, participants completed a written consent of participation. Each dyad carried out the sandwich-making task twice so that each participant acted as both customer and worker. The customer was instructed to request 15 ingredients for their sandwich. Participants kept their own count of the number of ingredients ordered, stopping when they had reached 15. The customer was further instructed to only request a single ingredient at a time and to refrain from directly pointing to or touching the ingredients. Upon completing the first sandwich, an experimenter entered the study room and reset the ingredients back to their original locations on the table, and the participants switched roles for the second sandwich.

Throughout the data collection study, both participants wore mobile eye-tracking glasses developed by SMI^1^. These eye-trackers perform binocular dark-pupil tracking with a sampling rate of 30 Hz and gaze position accuracy of 0.5°. Each set of glasses contains a forward-facing high-definition (HD) camera that was used to record both audio and video at 24 fps. The gaze trackers were time-synchronized with each other so that the gaze data from both participants could be correlated.

Following data collection, the proprietary BeGaze software created by SMI was used to automatically segment the gaze data into fixations—periods of time when the eyes were at rest on a single target—and saccades—periods of time when the eyes were engaged in rapid movement. Fixations were labeled with the name of the target fixated upon. Possible targets included the sandwich ingredients (Figure 1), the slices of bread, the conversational partner, and elsewhere in space. Speech was also transcribed for each participant. Customer requests for specific objects were tagged with the ID of the referenced object.

### 3.2. Intention modeling

In this work, we considered the customers' intentions to be their chosen ingredients. Informed by the literature, we hypothesized that the customers' gaze patterns would signify their intent of which ingredients they wanted on their sandwich and aimed to develop a model to accurately predict intentions based on their gaze patterns. Our data collection resulted in a total of 334 episodes of ingredient requests. We excluded episodes where more than 40% of the gaze data was missing before verbal requests, yielding 276 episodes for data analysis and modeling.

A naive, but plausible, strategy to predict a person's intent is solely based on his or her current gaze, which may indicate the person's current attention and interest (Frischen et al., 2007). To evaluate the efficacy of this strategy, we built an *attention-based* intention predictor that performed predictions according to which ingredient the customer most recently fixated on. An evaluation of the 276 episodes showed that the attention-based predictor achieved 65.22% accuracy in predicting the customers' choice of ingredient. This strategy outperformed random guesses of the ingredient, which were between 4.35 (i.e., 1/23) and 11.11% (i.e., 1/9), depending on how many potential ingredients were still available at that point in the interaction.

While the attention-based method was reasonably effective in predicting the intended ingredients, it only relied on the most recently glanced-at ingredient and omitted any prior gaze cues. However, the history of gaze cues may provide richer information for understanding and anticipating intent. In particular, we made two observations from the 276 episode analysis. First, participants seemed to glance at the intended ingredient longer than other ingredients. Second, participants glanced multiple times toward the intended ingredient before making the corresponding verbal request. These observations, along with significance of attention, informed our selection of characteristic features, as listed below, to represent patterns of participant's gaze cues. Each of the four features was computed for all potential ingredients in every episode of an ingredient request.

**Table:** 

Feature 1	Number of glances toward the ingredient before the verbal request (Integer)
Feature 2	Duration (in milliseconds) of the first glance toward the ingredient before the verbal request (Real value)
Feature 3	Total duration (in milliseconds) of all the glances toward the ingredient before the verbal request (Real value)
Feature 4	Whether or not the ingredient was most recently glanced at (Boolean value)

We applied a support vector machine (SVM) (Cortes and Vapnik, 1995)—a type of supervised machine learning approach that is widely used for classification problems—to classify the participants' gaze patterns into two categories, one for the intended ingredient (i.e., positive) and the other for the non-intended, competing ingredients (i.e., negative). In this work, we used Radial Basis Function (RBF) Kernels and the implementation of LIBSVM (Chang and Lin, 2011) for the analysis and evaluation reported below.

To evaluate the effectiveness of our model in classifying gaze patterns for user intentions, we conducted a 10-fold cross-validation using the 276 episodes of interaction. For each episode, we calculated a feature vector, including Features 1–4, for each ingredient that the customer looked toward before making a verbal request. To train the SVM, if an ingredient was the requested ingredient, the classification label was set to 1; otherwise, it was set to −1. In the test phase, the trained SVM determined the classification for each ingredient glanced at. On average, the SVMs achieved 89.00% accuracy in classifying labels of customer intention. Feature selection analyses (Chen and Lin, 2006) revealed that Feature 3 was the most indicative in classifying intentions, followed by Feature 4, Feature 1, and then Feature 2.

### 3.3. Intention prediction

The SVM classifier was further modified to predict the customers' intentions. The input to our SVM predictor was a stream of gaze fixations. As the interaction unfolded, we maintained a list of candidate ingredients, their corresponding feature vectors, and the estimated probabilities of the ingredient being the intended request, calculated using the method based on Wu et al. (2004). When a new gaze fixation on an ingredient occurred, we first checked whether or not the ingredient was in the candidate list. If the ingredient was already in the list, we updated its feature vector and estimated probability; otherwise, we added a new entry for the ingredient to the list.

A traditional SVM was used to classify an ingredient to be the potential request if the estimated probability was greater than 0.5. If more than one ingredient was classified as a potential request, the traditional SVM predictor picked the ingredient with the highest probability as the final prediction. If, however, none of the ingredients were classified as potential requests, the predictor made no prediction. The effectiveness of such a traditional SVM predictor was assessed via a 10-fold cross-validation using our 276 episodes. For this evaluation, a prediction was considered to be correct only when the prediction matched the actual request. Note that this intention prediction was different from the classification of gaze patterns reported in the previous section. The accuracy of intention prediction was assessed by whether or not the predicted ingredients matched the requested ones, whereas the accuracy of intention classification was based on comparisons of classified labels, including both positive and negative, with actual labels. The traditional SVM predictor on average reached 61.52% accuracy in predicting which ingredients the customer would pick. Further analysis revealed that 28.99% of the time the SVM predictor made no predictions. However, when it made predictions (i.e., 71.01% of the time), the SVM provided predictions at 86.43% accuracy. This accuracy could be interpreted as the confidence of the traditional SVM predictor in predicting intention when it had a positive classification.

**Figure 2:**
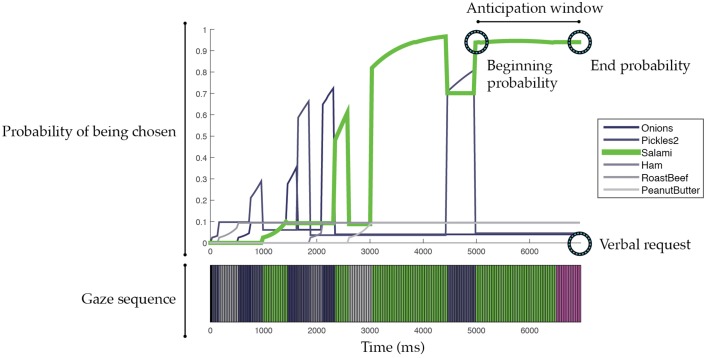
**Illustration of episodic prediction analysis**. Each illustrated episode ends at the start of the verbal request. The top plot shows probabilities of glanced ingredients that may be chosen by a customer. Note that the plotted probability was with respect to each ingredient. By calculating the normalized probability across all ingredients, we can determine the likelihood of which ingredient will be chosen. The bottom plot shows the customer's gaze sequence. Ingredients are color coded. Purple indicates gazing toward the bread. Black indicates missing gaze data. An anticipation window is defined as the time period starting with the last change in the prediction and ending with the onset of the speech utterance. The beginning and end probabilities are the probabilities of the predicted ingredient at the beginning and end of the anticipation window.

We defined an anticipation window as the time period starting with the last change in the prediction and ending with the onset of the speech utterance (see Figure 2 as an example). This anticipation window allowed us to understand how early the predictor could reach the correct predictions. For the traditional SVM predictor, the anticipation window for the correct predictions was on average 1420.57 ms before the actual verbal request, meaning that the predictor could anticipate the intended ingredient about 1.4 s in advance. The interaction duration before the verbal request for the episodes with correct predictions was on average 3802.56 ms (*SD* = 1596.45).

The predictive accuracy of the traditional SVM predictor was largely impaired by the frequency with which it made no predictions. To address this issue, we ensured that our SVM-based predictor always made a prediction, choosing the ingredient with the highest probability. A 10-fold cross-validation using the 276 episodes showed that our SVM-based predictor on average reached 76.36% predictive accuracy and could make those correct predictions 1831.27 ms ahead of their corresponding verbal requests (Interaction duration *M* = 3802.56, *SD* = 1596.45). Table 1 summarizes these results. Moreover, we analyzed the probabilities of the chosen ingredients that were at the beginning and end of the anticipation window (see Figure 2). On average, the beginning and end probabilities for the correct predictions were 0.36 and 0.75, respectively, whereas the beginning and end probabilities for the incorrect predictions were 0.28 and 0.43, respectively. These probability parameters indicate the confidence of our SVM-based predictor in making a correct prediction. For example, when the probability of an ingredient is over 0.43, the ingredient is likely to be the intended choice. We note that this threshold (0.43) is lower than the threshold used by the traditional SVM (0.50). Similarly, if the probability of an ingredient is lower than 0.36, the ingredient is less likely to be the intended choice. These parameters allow the construction of a real-time intention predictor that anticipates the customers' choices on the fly.

**Table 1:** **Summary of our quantitative evaluation of the effectiveness of different intention prediction approaches**.

	**Predictive accuracy**	**Anticipation time**
Chance	4.35–11.11%	N/A
Attention-based	65.22%	N/A
SVM-based	76.36%	1831 ms

In the next section, we provide examples and further analyses of when our SVM-based predictor made correct and incorrect predictions. These analyses revealed gaze patterns that may provide additional insight into understanding the customers' intentions.

### 3.4. Qualitative analysis

To further understand how our intention predictor made correct and incorrect predictions in the collected interaction episodes, we plotted the probability of each glanced-at ingredient over time, aligned with the corresponding gaze sequence received from the gaze tracker, for each interaction episode (see Figure 2 for an example). These plots facilitated a qualitative analyses of gaze patterns and further revealed patterns that were not captured in our designed features but may signify user intentions. In the following paragraphs, we present our analyses and discuss exemplary cases.

#### 3.4.1. Correct predictions

**Figure 3:**
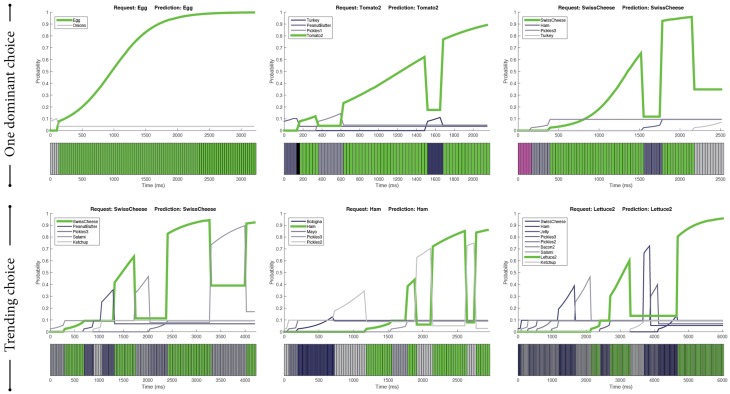
**Two main categories of correct predictions: one dominant choice (top) and the trending choice (bottom)**. Green indicates the ingredients predicted by our SVM-based predictor that were the same as the actual ingredients requested by the customers. Purple indicates gazing toward the bread and yellow indicates gazing toward the worker. Black indicates missing gaze data.

Two categories—one dominant choice and the trending choice—emerged from the episodes with correct predictions (see examples in Figure 3).

##### 3.4.1.1. One dominant choice

In this category, customers seemed to be focused toward one dominant ingredient, which was apparent in their gaze cues (Figure 3, Top). In particular, we found two types of gaze patterns. In the first, participants looked toward the intended ingredient for a prolonged time. In the second, they looked toward the intended ingredient multiple times in the course of their interaction. For both patterns, the intended ingredient received the majority of the gaze attention relative to other ingredients. This dominance allowed the predictor to give correct predictions.

##### 3.4.1.2. Trending choice

In contrast to the previous category, there were situations in which customers did not seem to have a single ingredient in mind. In these situations, the customers exhibited a “shopping” behavior by looking toward multiple ingredients to decide which one to order. These situations usually involved the participants' visual attention being spread across multiple candidate ingredients. However, the customers generally looked toward the intended ingredient recurrently compared to other competing ingredients throughout the interaction. This recurrent pattern resulted in the intended ingredient becoming a trending choice, as illustrated in the bottom examples of Figure 3. The SVM-based predictor was observed to capture this pattern effectively.

#### 3.4.2. Incorrect predictions

From the 10-fold evaluation of the SVM-based predictor, there were a total of 62 episodes resulting in incorrect predictions. In the following paragraphs, we describe the characteristics of four identified categories of these incorrect predictions.

##### 3.4.2.1. No intended glances

**Figure 4:**
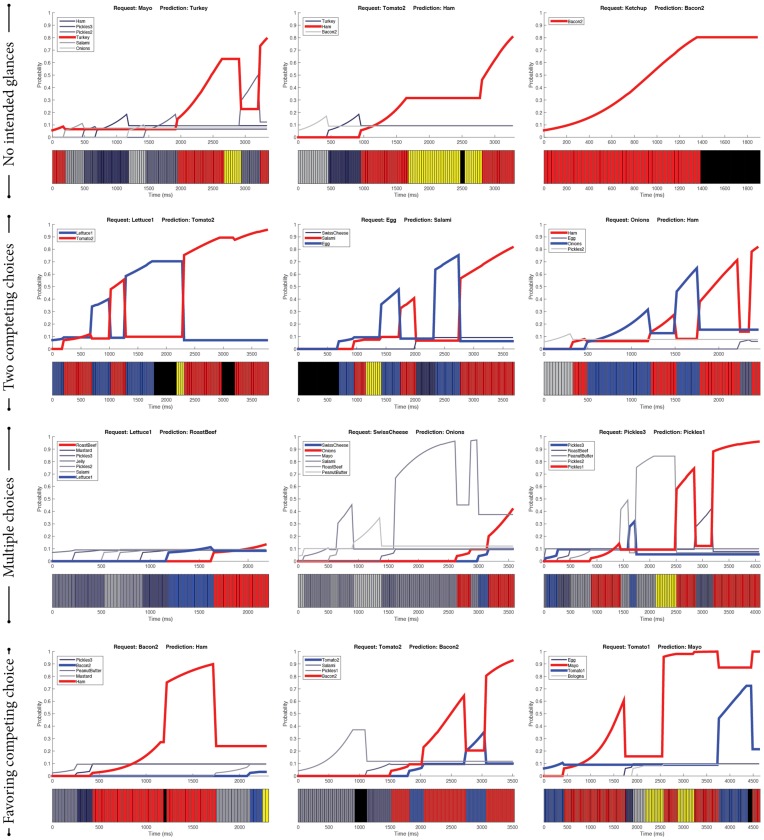
**Examples of incorrect predictions**. Red indicates the prediction made by the SVM-based predictor, whereas blue indicates the actual ingredient requested by the customers. Purple indicates gazing toward the bread whereas yellow indicates gazing toward the worker. Black indicates missing gaze data.

Among the incorrect predictions, there were 23 episodes (37.10%) during which the customers did not glance at the intended ingredients (Figure 4, First row). There are three reasons that might explain these cases. First, the customers had made their decisions in previous episodes. For example, when they were glancing around to pick an ingredient, they may have also decided which ingredient to order next. Second, their intentions were not explicitly manifested through their gaze cues. Third, the gaze tracker did not capture the gaze of the intended ingredient (i.e., missing data). In each of these cases, the predictor could not make correct predictions as it did not have the necessary information about the intended ingredients.

##### 3.4.2.2. Two competing choices

Sometimes, customers seemed to have two ingredients they were deciding between (Figure 4, Second row). In this case, their gaze cues were similarly distributed between the competing ingredients. Therefore, gaze cues alone were not adequate to anticipate the customers' intent. We speculate that the determinant factors in these situations were subtle and not well-captured via gaze cues. Therefore, the predictor was likely to make incorrect predictions in these situations.

##### 3.4.2.3. Multiple choices

Similar to the case of two competing choices, the customers sometimes decided among multiple candidate ingredients (Figure 4, Third row). As gaze cues were distributed across candidate ingredients, our predictor had difficulty in choosing the intended ingredient. Additional information, either from different behavioral modalities or new features of gaze cues, is necessary to distinguish the intended ingredient from the competing ones.

##### 3.4.2.4. Favoring competing choices

In situations where the customers looked toward competing ingredients more frequently as compared to the intended ingredient, our predictor made incorrect predictions (see examples in Figure 4, Fourth row). One potential explanation for this type of gaze pattern is that the customers changed their decision after quick glances at the intended ingredients. For instance, as shown in the bottom examples of Figure 4, while the customers looked longer and multiple times at the red ingredient, they requested the blue ingredient with smaller gaze attention. Our features failed to capture such quick decisions, likely resulting in incorrect predictions.

#### 3.4.3. Special patterns

In analyzing the efficacy of our SVM-based intention predictor, we observed some special, potentially informative gaze patterns that were not explicitly captured in our derived features emerge. We discuss these patterns in the following paragraphs.

##### 3.4.3.1. Initiating joint attention

**Figure 5:**
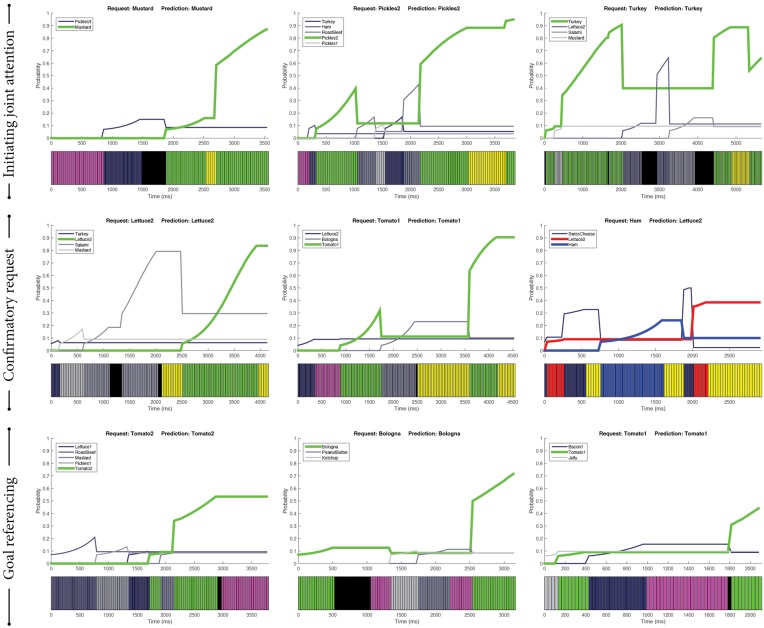
**Examples of special gaze patterns**. Green indicates the ingredients predicted by our SVM-based predictor that were the same as the actual ingredients requested by the customers. Blue lines indicate the ingredients that the customers picked. Red lines are our predictions. Purple indicates gazing toward the bread, whereas yellow indicates gazing toward the worker. Black indicates missing gaze data.

Initiating joint attention is the process of using behavioral cues to direct the other's attention to a shared artifact. One such behavioral instantiation involves alternating gaze cues—looking toward the intended ingredient, looking toward the worker, and then looking back at the intended ingredient (Mundy and Newell, 2007). We found such patterns of initiating joint attention in our data, as shown in the first row of Figure 5. This pattern usually emerged toward the end of the episode, serving as a signal to the worker that the intended ingredient had been chosen.

##### 3.4.3.2. Confirmatory request

The inverse pattern of initiating joint attention is that of the customer looking toward the worker, toward the intended ingredient, and then back toward the worker. Conceptually, we can characterize this pattern as a confirmatory request, meaning that the customer sought the worker's attention, directed their attention, and checked if the intention was understood. From our data, this pattern of confirmatory request seemed to signify intention. As illustrated in the second row of Figure 5, the single ingredient between fixations at the worker was the intended ingredient.

##### 3.4.3.3. Goal referencing

Another pattern that emerged from the data was visual references to the goal, which in our context was the bread where ingredients were moved. This type of reference was found in a variety of combinations. It could be found before, after, or in between choosing the intended ingredient. Examples are provided in the third row of Figure 5. There may be different meanings to these combinations. For instance, the customers might have checked which ingredients had been added to the sandwich and used that information to decide which ingredient to pick next.

## 4. Discussion

To quantitatively investigate the relationship between exhibited gaze cues and intentions, we contextualized our investigation in a sandwich-making scenario in which a worker made a sandwich using ingredients requested by a customer. We characterized intentions as the ingredients requested by the customers and hypothesized that the customers' gaze patterns would predict their choice of ingredients. We developed an SVM-based intention predictor using four features that aimed to represent characteristics of the customers' gaze patterns. The SVM-based predictor was demonstrated to outperform the basic attention-based predictor in predicting the customers' choices of ingredients. Moreover, the SVM-based predictor could make correct predictions approximately 1.8 s before the requests. Furthermore, we qualitatively analyzed the instances of correct and incorrect predictions made by the SVM-based predictor to better understand its performance in boundary cases. In this section, we discuss implications of our qualitative analyses, potential applications of our intention predictor, and limitations of the present work.

### 4.1. Implications of qualitative analyses

Our qualitative analyses (Section 3.4) provided not only insight into how the SVM-based predictor made correct and incorrect predictions, but they also revealed special patterns that may *signal* intentions via visual references to the other person and the goal. Signaling is an intentional strategy that people use to manifest actions and intentions in a way that is more predictable and comprehensible to interaction partners (Pezzulo et al., 2013). For example, parents exaggerate intonation in infant-directed speech (Kuhl et al., 1997). The use of signaling strategies facilitates the formation of common ground. The special patterns *initiating joint attention* and *confirmatory request* involved interleaving gaze cues between the partner and the intended ingredient. These displays of interleaving gaze may serve as an intentional signaling strategy, highlighting the relevance of the intended ingredient. Similarly, the visual references to the goal, which is the bread in our scenario, may be signaling the intentional link between the bread and the intended ingredient, as shown in the pattern *goal referencing*.

The four features of gaze cues explored in this work were based on statistical measures of the customers' gaze sequences. While these features seemed to capture how the distribution of gaze cues may indicate intentions, they did not explicitly encode sequential structures from gaze sequences. However, sequential structures—such as gaze toward the target, then partner, and then the target again—may encapsulate particular semantic meanings, such as directing the partner's attention toward the target. The capability to recognize these sequential structures as those of *initiating joint attention, confirmatory request*, and *goal referencing*, could reveal the underlying meanings of gaze sequence and potentially improve the efficacy of the SVM-based predictor. For example, the last plot of the examples of *confirmatory request* showed that the intention predictor could benefit from recognizing the sequential human-target-human pattern. One way to recognize such sequential structures is through template matching, which has been explored to recognize communicative backchannels (Morency et al., 2010).

However, the special patterns, identified in Section 3.4.3, should be used with caution when predicting intentions. The last plot in Figure 4 illustrated a contradictory example; even though there was a clear pattern of *confirmatory request*, it did not signify the intended ingredient. Further research is necessary to investigate how the incorporation of sequential structures into the predictive model may enhance predictive performance.

### 4.2. Applications

The capability to interpret others' intentions and anticipate actions is critical in performing joint actions (Sebanz and Knoblich, 2009; Huber et al., 2013). Prior research has explored how reading intention and performing anticipatory actions might benefit robots in providing assistance to their users, highlighting the importance of intention prediction in joint actions between humans and robots (Sakita et al., 2004; Hoffman and Breazeal, 2007). Building on prior research, this work provides empirical results showing the relationship between gaze cues and human intentions. It also presents an implementation of an intention predictor using SVMs. With the advancement of computing and sensing technologies, such as gaze tracking systems, we anticipate that an even more reliable intention predictor could be realized in the foreseeable future. Computer systems such as assistive robots and ubiquitous devices could utilize intention predictors to augment human capabilities in many applications. For example, robot co-workers could predict human workers' intentions by monitoring their gaze cues, enabling the robots to choose complementary tasks to increase productivity in manufacturing applications. Similarly, assistive robots could provide necessary assistance to people by interpreting their gaze patterns that signal intended help. In addition to applications involving physical interactions, recommendation systems could provide better recommendations to users by utilizing their gaze patterns. For instance, an online shopping website could dynamically recommend products to customers by tracking and interpreting their gaze patterns.

### 4.3. Limitations

The current work also has limitations that motivate future investigations. First, we employed SVMs for data analysis and modeling to quantify the potential relationship between gaze cues and intentions. Alternative approaches, such as decision trees and hidden Markov models (HMMs), may also be used to investigate such relationships and interaction dynamics. However, similar to most machine learning approaches that are sensitive to the data source, our results were subject to the interaction context and the collected data. For instance, the parameters of the predictive window (e.g., size) might be limited to our present context. Yet, in this work, we demonstrated that characteristics of gaze cues, especially duration and frequency, are a rich source for understanding human intentions. Furthermore, we used a toy set of sandwich items as our research apparatus. Participants working with the toy sandwich may have produced different gaze patterns then they would when working with real sandwich materials.

Second, we formulated the problem of intention prediction in the context of sandwich-making as the problem of using the customers' gaze patterns to predict their choices of ingredients. Intention is a complex construct that may not be simply represented as the requested ingredient. While our work focused solely on using gaze cues to predict customer intent, workers in this scenario may rely on additional features, including facial expressions and other cues from the customer, and other forms of contextual information, such as preferences expressed previously toward particular toppings or knowledge of what toppings might “go together.” Disentangling the contributions of different features to observer performance in these predictions would significantly enrich our understanding of the process people follow to predict intent. However, our findings were in line with literature indicating that gaze cues manifest attention and lead intended actions (Butterworth, 1991; Land et al., 1999; Johansson et al., 2001). In addition, the sequences of gaze cues, as inputs to our predictive model, were obtained via a gaze tracker worn by the customers. Future research may consider acquiring the gaze sequences from the perspective of the worker. This approach may be beneficial in developing an autonomous robotic assistant (Ognibene and Demiris, 2013; Ognibene et al., 2013) that can leverage its onboard camera to obtain the different items human users gaze toward. Future work may also compare the performance of human observers and the types of errors they make to those of our machine learning model. Such a comparison may inform our selection of features or learning algorithms in building systems that recognize user intent.

## 5. Conclusion

Eye gaze is a rich source for interpreting a person's intentions. In this work, we developed a SVM-based approach to quantify how gaze cues may signify a person's intention. Using the data collected from a sandwich-making task, we demonstrated the effectiveness of our approach in a laboratory evaluation, where our predictor provided improved accuracy in making correct predictions of the customers' choices of ingredient (76%) compared to the attention-based approach (65%) that only relied on the most recently glanced-at ingredient. Moreover, our SVM-based approach provided correct predictions approximately 1.8 s before the requests, whereas the attention-based approach did not afford such intention anticipation. Analyses of the episodic interactions further revealed gaze patterns that suggested semantic meanings and that contributed to correct and incorrect predictions. These patterns informed the design of gaze features that offer a more complete picture of human intentions. Our findings provide insight into linking human intentions and gaze cues and offer implications for designing intention predictors for assistive systems that can provide anticipatory help to human users.

### Conflict of interest statement

The authors declare that the research was conducted in the absence of any commercial or financial relationships that could be construed as a potential conflict of interest.

## Footnotes

^1^http://www.smivision.com/en/gaze-and-eye-tracking-systems/home.html
